# Icariin Prevents IL-1*β*-Induced Apoptosis in Human Nucleus Pulposus via the PI3K/AKT Pathway

**DOI:** 10.1155/2017/2198323

**Published:** 2017-11-12

**Authors:** Xiangyu Deng, Wei Wu, Hang Liang, Donghua Huang, Doudou Jing, Dong Zheng, Zengwu Shao

**Affiliations:** ^1^Department of Orthopaedics, Union Hospital, Tongji Medical College, Huazhong University of Science and Technology, Wuhan 430022, China; ^2^Department of Pediatrics, Tongji Hospital, Tongji Medical College, Huazhong University of Science and Technology, Wuhan 430022, China

## Abstract

**Purpose:**

To explore the effect and possible mechanism of icariin, a prenylated flavonol glycoside derived from the Chinese herb* Epimedium sagittatum *that was applied to IL-1*β* pretreated human nucleus pulposus (NP) cells.

**Methods:**

Human NP cells were isolated from intervertebral discs of patients with scoliosis and lumbar spondylolisthesis. The cells were divided into five groups: A (blank control); B (20 ng/ml IL-1*β*); C (20 ng/ml IL-1*β* + 20 *μ*M icariin); D (20 *μ*M icariin + 20 ng/ml IL-1*β* + 25 *μ*M LY294002); E (20 ng/ml IL-1*β* + 25 *μ*M LY294002). For each of the five groups, the CCK8, apoptosis rates, ROS rates, and JC-1 rates were determined and an electron micrograph was performed. Different expression levels of apoptosis proteins and proteins in the PI3K/AKT pathway were detected via western blot.

**Results:**

We found that the damage effects on human nucleus pulposus cells from 20 ng/ml of IL-1*β* exposure were attenuated by icariin. When the PI3K/AKT pathway was blocked by LY294002, a specific inhibitor of this pathway, the protective effect of icariin was impaired. In summary, icariin might be a protective traditional Chinese medicine, which prevents inflammation-induced degeneration of intervertebral discs partly through the PI3K/AKT pathway.

## 1. Introduction

Inflammation is involved in many pathological processes and is associated with the degeneration of intervertebral disc [1]. Nucleus pulposus, located in the center of intervertebral discs, lacks a blood supply, oxygen, and nutrition [2]. It is often exposed to an inflammatory microenvironment. IL-1*β*, a proinflammatory cytokine involved in inflammatory processes and the induction of apoptosis in response to cell injury, has been reported to have a connection with the degeneration of intervertebral discs [3]. Low back pain (LBP) is a frequent musculoskeletal disorder worldwide that affects approximately 70% of the adult population, sometime during their lives, and frequently results in musculoskeletal disability. According to statistics [4–8], LBP can exert an enormous economic burden every year.

Icariin is the most frequently used medicinal herb in traditional Chinese medicine. This micromolecule steroid compound is extracted from herba epimedii and is mostly applied in the treatment of osteoporosis, especially in postmenopausal osteoporosis. What is more, icariin has also been reported to have protective effects against amyloid beta-induced apoptosis in PC-12 cells [9], attenuating LPS-induced acute inflammatory responses [10], and protecting against MPP(+)-induced toxicity in MES23.5 cells [11]. Additionally, several complex roles for icariin in a number of pathway activation instances have been proposed, such as the PI3K/AKT pathway [9, 10, 12–15], the ROS/JNK-dependent mitochondrial pathway [16], the Wnt/beta-catenin pathway [17], and NO/cGMP signaling [18].

In this work, we simulated an inflammatory activation microenvironment in the intervertebral disc through the use of IL-1*β* at 20 ng/ml. We try to explore the protective effect of icariin on IL-1*β*-induced inflammatory intervertebral disc model. We also used LY294002, an inhibitor of the PI3K/AKT pathway, in order to verify the PI3K/AKT pathway participating in the anti-inflammatory function of icariin.

## 2. Materials and Methods

### 2.1. General Supplies

Instruments, reagents, and the experimental animals were provided by the animal center of Tongji Medical College and Huazhong University of Science and Technology. IL-1*β* was purchased from Thermo Fisher Scientific (Waltham, MA, USA). Icariin (purity ≥ 98%) was purchased from Nanjing Zelang Pharmaceutical Technology (Nanjing, China). Fetal bovine serum was purchased from Gibco. F12-Dulbecco's modified Eagle medium was purchased from Hyclone (Logan, UT, USA). Cell counting kit-8 (CCK8) was purchased from Kaiji Bioengineering Institute (Jiangsu, China). LY294002 was purchased from Sigma-Aldrich (St. Louis, MO, USA). The reactive oxygen species (ROS) detection kit was purchased from Nanjing Jiancheng Bioengineering Institute (Nanjing, China). JC-1 assay kit was purchased from Beyotime (Beijing, China). Annexin V-FITC/propidium iodide detection kit was purchased from Nanjing KeyGen Biotech (Nanjing, China). *β*-Actin, Bcl-2, bax, caspase-3, phospho(p)-AKT, rabbit monoclonal antibodies, and the p53 and AKT mouse monoclonal antibody were purchased from Abcam (Cambridge, UK). Goat antirabbit and goat antimouse IgG were purchased from Proteintech (Wuhan, China). Microplate reader was purchased from Thermo Fisher Scientific (Waltham, MA, USA). The inverted fluorescence microscope used was manufactured by Olympus (Japan).

### 2.2. Culture and Synchronization of the NP Cells and the Detection of Cell Density and Morphology [19]

The density and morphology of NP cells under different treatments were observed and photographed with an inverted phase contrast microscope. NP cells were isolated from the nucleus pulposus tissue from a patient that underwent surgery for scoliosis. Briefly, NP tissue was aseptically removed, placed in a Petri dish containing 0.25% (w/v) type II collagenase, and cut into 0.1 mm × 0.1 mm pieces. Samples were digested with 0.25% (w/v) type II collagenase overnight and serum was used to stop the reaction. After centrifugation at 1200 rpm for 7 min, the supernatant was discarded and the pellet was resuspended in F12-Dulbecco's modified Eagle medium, supplemented with 20% fetal bovine serum, 100 U/mL penicillin, and 100 mg/L streptomycin. Cell cultures were maintained at 37°C and 5% CO_2_. The medium was changed 3–5 days later, when the cells had been attached, and then changed every other day after that. When NP cells reached approximately 80% confluence, each primary culture was subcultured at a 1 : 3 ratio with a 0.25% (w/v) trypsin solution.

### 2.3. Experimental Protocols

Cells were tested for the ability of icariin to activate the PI3K/AKT pathway. Kinetics of the phosphorylation of AKT were estimated by western blot analysis at 0 h, 1 h, 2 h, 3 h, 4 h, and 5 h. The remaining cells were randomly separated into five groups with at least three replicates: A (blank control); B (20 ng/ml IL-1*β*); C (20 ng/ml IL-1*β* + 20 *μ*M icariin); D (20 *μ*M icariin + 20 ng/ml IL-1*β* + 25 *μ*M LY294002); E (20 ng/ml IL-1*β* + 25 *μ*M LY294002). Treatment with LY294002, icariin, and IL-1*β* was performed for 2 h, 24 h, and 48 h, respectively. Icariin and LY294002 were both preporcessed. That means we added LY294002 in the medium for 2 h and then took it out by transferring the medium. Icariin was then added for 24 h and then removed. In the end, IL-1*β* was added for 48 h and different detection was conducted.

### 2.4. Detection of Icariin Cytotoxicity, Cell Viability, and Proliferation

The cytotoxicity of cells exposed to icariin treatments was evaluated by measuring lactate dehydrogenase (LDH) release using a CytoTox96® Non-Radioactive Cytotoxicity Assay kit (Promega), according to the manufacturer's instructions.

When exposed to different concentration of icariin, the cell viability was detected by CCK8 assay. NP cells at passage 3 were replated in 96-well plates at a density 1 × 10^5^ cells per well, and the culture medium was plated after synchronization. Cells were then treated with icariin for 24 h at various concentrations (0.1, 0.5, 1, 5, 10, 20, 40, and 50 *μ*M) to evaluate the effect of icariin on cell's proliferation rate. Cell viability was detected according to the instructions of the CCK8 assay. Then cells were treated according to the aforementioned experimental groupings. Cell viability was again detected according to the manufacturer's instructions.

### 2.5. Apoptosis Assay [19]

Cells were harvested and washed with PBS twice at 4°C. Next, cells were resuspended in 200 *μ*L of binding buffer and incubated with 10 *μ*L of Annexin V-FITC solution (15 min, room temperature) in the dark. Then cells were incubated with 10 *μ*L PI and 300 *μ*L binding buffer and immediately analyzed in a BD FACSCalibur cytometer to separate living cells, apoptotic cells, and necrotic cells into different periods.

### 2.6. Observation by Transmission Electron Microscope

The cells were double-fixed by glutaraldehyde and osmic acid, dehydrated by gradient acetone, immersed in embedding medium, ultrathin-sectioned using an automatic microtome (LeicaRM2235, Leica, Germany), and stained with 1% uranyl acetate. The cells' sections were observed and filmed under a transmission electron microscope (Hitachi, Japan) to observe the status of mitochondria in the human NP cells.

### 2.7. Mitochondrial Membrane Potential

Changes in the mitochondrial membrane potential were monitored using a JC-1 assay kit (Beyotime, Beijing, China), according to the manufacturer's instructions. Purified mitochondrial pellets (0.1 mL), with a total protein content of 100 *μ*g, were incubated with 0.9 mL of JC-1 dye working solution for 20 min, and the fluorescence intensity was immediately measured using a fluorescence spectrophotometer (Shimadzu RF 5301, Kyoto, Japan). In the mitochondria with high membrane potential, the JC-1 dye mainly existed in the mitochondrial matrix with red fluorescent aggregates, while green fluorescence represented the monomeric form of JC-1 and the mitochondria with low membrane potential. The red/green fluorescence intensity ratio was used to denote the level of mitochondrial membrane potential depolarization (Ex = 525 nm and Em = 590 nm for aggregates; Ex = 490 nm and Em = 530 nm for JC-1 monomers).

### 2.8. Detection of Intracellular ROS Levels by Flow Cytometry

Cells were treated according to the aforementioned experiment grouping design. Then, 200 *μ*L of culture medium from each group was collected to detect intracellular ROS levels. Experimental steps were strictly executed according to the manufacturer's instructions.

### 2.9. Expression of AKT, p-AKT, p53, Bcl-2, Bax, and Caspase-3 by Western Blot Analysis

Proteins were extracted according to the instructions of the Total Extraction Sample Kit. Equal amounts of the proteins (10 *μ*g) were loaded onto 10% sodium dodecyl sulfate polyacrylamide gels, electrophoresed, and then transferred to polyvinylidene fluoride membranes. The membranes were incubated with 5% nonfat milk for 2 h followed by incubation with primary antibodies overnight at 4°C (0.5 *μ*g/mL AKT, p53, p-AKT, Bcl-2, and caspase-3; 1 : 5000). After washing in TBST, membranes were incubated with the secondary antibody for 1.5 h at room temperature (rabbit anti-mouse or goat anti-rabbit, 1 : 5000). Bands were visualized by incubating with an enhanced chemiluminescent reagent for 2 min after the membranes were washed with TBST. Densitometry measurements of p-AKT, AKT, Bcl-2, bax, and caspase-3 levels were performed using Image J software (National Institutes of Health, Bethesda, MD, USA).

### 2.10. Statistical Analysis

Data are presented as means ± standard deviation. For group-wise comparisons, a one-way ANOVA with the LSD or Dunnett's T3 test was performed using SPSS 19.0 (IBM, Chicago, IL, USA). Values were considered significantly different at ^*∗*^*p* < 0.05.

## 3. Results

### 3.1. Human Nucleus Pulposus Cells Were Successfully Separated and Cultured (The Cell Photograph Was Shown in Figure 1(a))

**Figure 1:**
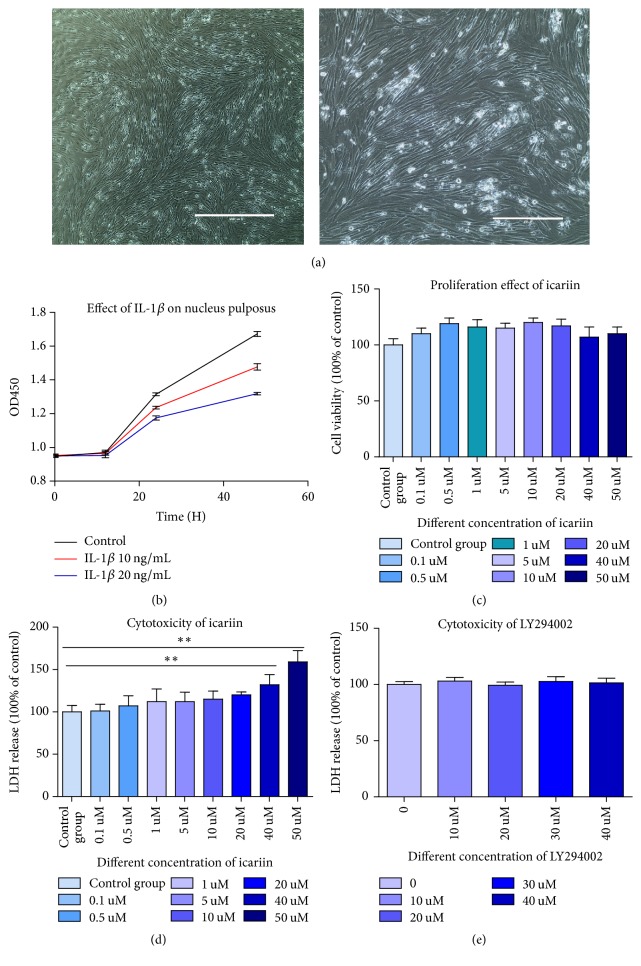
(a) Human NP cells showed a long spindle shape and exhibited good growth. (b) IL-1*β* retarded human nucleus pulposus growth over time and depending on its concentration. (c) Icariin has no promoting or inhibiting effects on cell proliferation at the concentration of 0.1 uM to 20 uM. (d) There was no cytotoxicity of icariin on cell at the concentration of 0.1 uM to 40 uM. When its concentration reached 40 uM, the cell membrane was observed to be unstable. There was significant difference between control group and 40 uM and 50 uM (^*∗∗*^*p* < 0.01).

IL-1*β* at concentration of 10 ng/ml and 20 ng/ml both delayed the grow rate in human nucleus pulposus cells and as the concentration goes up, inhibiting effect has been strengthened. We used the concentration of 20 ng/ml for follow-up experiment for its strong effect of inhibition and damage. We found that, over time, nucleus pulposus cells treated with IL-1*β* grew slower compared to the control group. Results were shown as a growth curve in Figure 1(b). What is more, we found that icariin had no promoting or inhibiting effect in cell proliferation at the concentration of 0.1 uM to 50 uM but when concentration reached 50 uM, cytotoxicity could be detected by LDH release assay. Results were shown in Figures 1(c) and 1(d). What is more, LY294002 has no cytotoxicity at working concentration of 25 uM with the time. Results were shown in Figure 1(e).

### 3.2. Icariin Decreased IL-1*β*-Induced Apoptosis Rate in Human Nucleus Pulposus Cells

**Figure 2:**
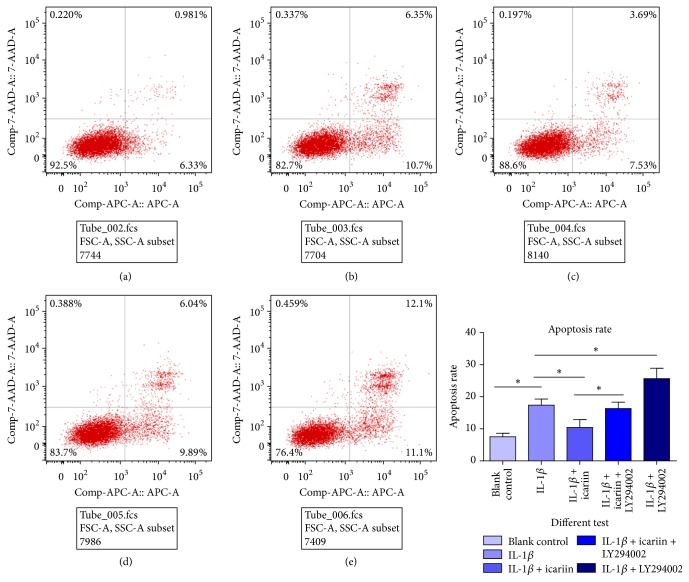
(a) Blank control; (b) 20 ng/ml IL-1*β*; (c) 20 ng/ml IL-1*β* + 20 *μ*M icariin; (d) 20 *μ*M icariin + 20 ng/ml IL-1*β* + 25 *μ*M LY294002; (e) 20 ng/ml IL-1*β* + 25 *μ*M LY294002. Compared with group A, IL-1*β* could induce apoptosis in human NP cells. When cells were pretreated with icariin, the apoptosis rate decreased. However, we noted that the PI3K/AKT pathway was involved in this protective effect, as the apoptosis rate increased when the PI3K/AKT pathway was blocked. All results were statistically significant (^*∗*^*p* < 0.05).

Apoptosis rate was detected by flow cytometry as shown in Figure 2. when IL-1*β* was added to the culture medium, human NP cells died at a significantly higher rate (Figures 2(a) and 2(b)) (^*∗*^*p* < 0.05). Pretreated with 20 *μ*M icariin for 24 h, the apoptosis rate decreased significantly (Figure 2(c)) (^*∗*^*p* < 0.05). When the PI3K/AKT pathway was blocked by LY294002, this protective effect was attenuated (Figure 2(d)) (^*∗*^*p* < 0.05). What is more, we found that if LY294002 was pretreated alone, compared with group B (20 ng/mL IL-1*β*), group E (20 ng/ml IL-1*β* + 25 *μ*M LY294002) showed higher apoptosis rates (Figure 2(e)) (^*∗*^*p* < 0.05). We thought LY294002 could be considered a risk factor alone.

### 3.3. Icariin Could Attenuate IL-1*β*-Induced Intercellular ROS Accumulation in Human Nucleus Pulposus Cells as Shown in Figure 3

**Figure 3:**
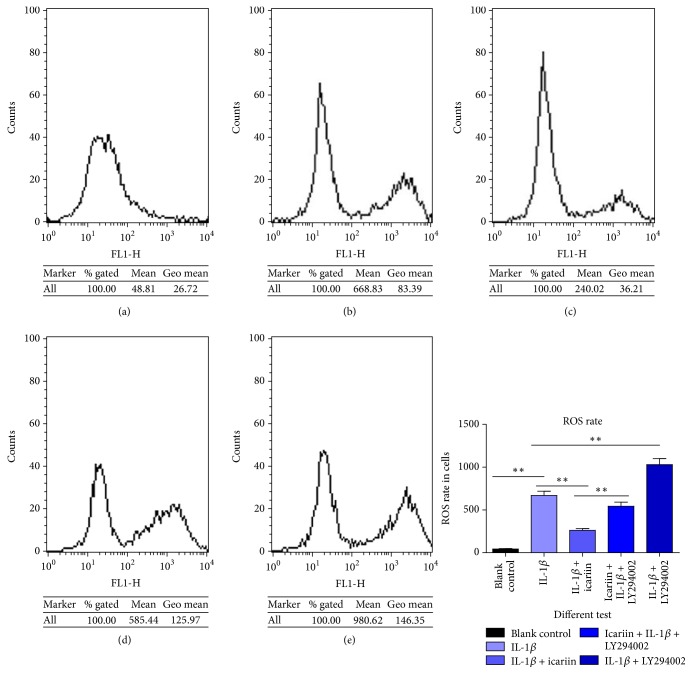
(a) Blank control; (b) 20 ng/ml IL-1*β*; (c) 20 ng/ml IL-1*β* + 20 *μ*M icariin; (d) 20 *μ*M icariin + 20 ng/ml IL-1*β* + 25 *μ*M LY294002; (e) 20 ng/ml IL-1*β* + 25 *μ*M LY294002. Intercellular ROS rates increased when human NP cells were exposed to 20 ng/mL IL-1*β*. Icariin attenuated the effect of IL-1*β*, to a certain extent. When PI3K/AKT pathway was blocked by LY294002, the protective effect of icariin was weakened. What is more, LY294002 could be an independent damage factor or had a synergistic effect with IL-1*β* to raise the intercellular ROS rate.

Intercellular ROS rates could reflect an oxidative stress status and were closely related to intercellular inflammation. ROS plays a key role in the inflammatory and cell damage processes. We observed that IL-1*β* induced the increase of intercellular ROS rate (Figures 3(a) and 3(b)), while icariin attenuated the damage (Figure 3(c)). The PI3K/AKT pathway was involved in this process (Figures 3(d) and 3(e)) (^*∗∗*^*p* < 0.01 versus control group).

### 3.4. Icariin Attenuated IL-1*β*-Induced Mitochondrial Membrane Potential (MMP) Losses (Figure 4)

The analysis of changes in MMP demonstrated that occur during apoptosis provides important information on the mechanisms and pathways of cell death. Changes in mitochondrial membrane potential were considered to be an early indicator of apoptosis. Variation in MMP could reflect the membrane state of cells.

### 3.5. Icariin Protects Human NP Cells from IL-1*β*-Induced Mitochondria Damage (Figure 5)

**Figure 5:**
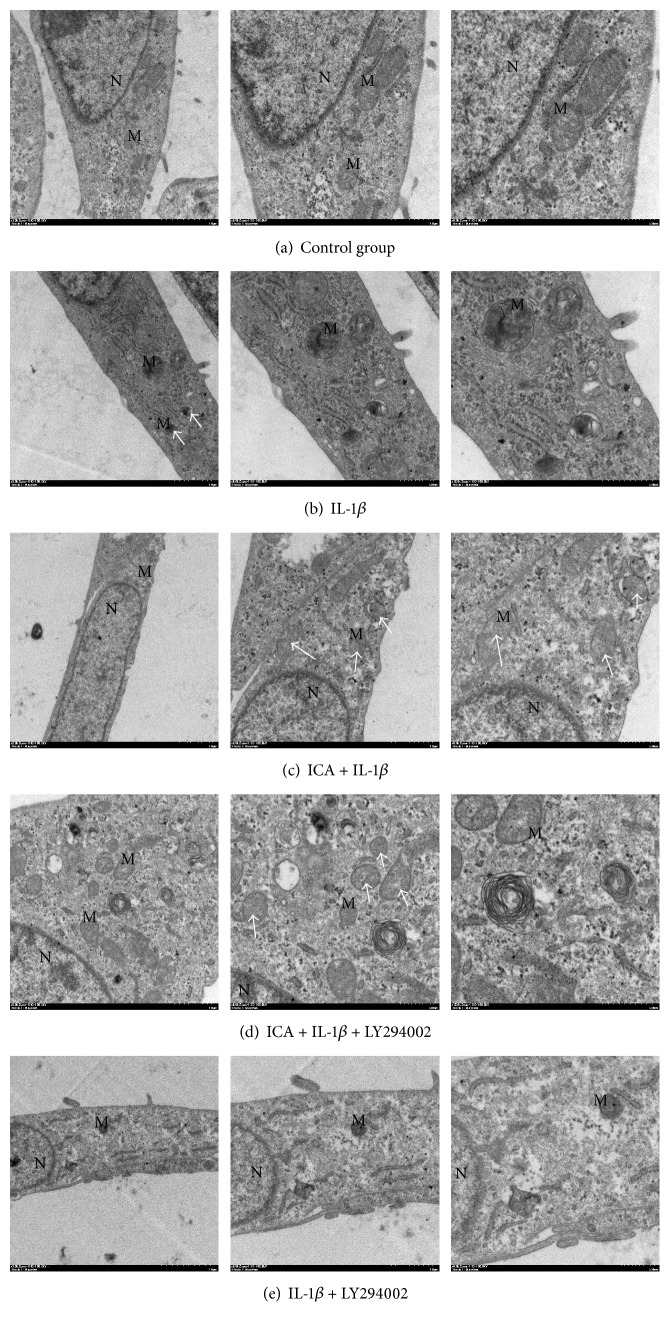
As shown above, IL-1*β* could induce mitochondrial swelling and membrane breakup and icariin could protect cells from this damage. LY294002 could attenuate the protecting effect and be an independent damage factor in the process of IL-1*β*-induced mitochondria damage.

Using transmission electron microscopy, we found IL-1*β* could induce mitochondrial swelling and membrane breakup (Figure 5(a)). In icariin pretreated cells, the damage was attenuated and protective effects were weakened by LY294002 (Figures 5(b)–5(d)). Compared to group B, group E sustained the most damage (Figure 5(e)), which indicated an independent injury function of LY294002.

### 3.6. Icariin Influences Apoptosis-Related Proteins, including Apoptosis Protein Bax, Caspase-3, and Antiapoptosis Protein Bcl-2 (Figure 6)

The result of western blot showed that icariin has a strong antiapoptosis effect on human NP cells, when cells were exposed to an inflammatory environment (^*∗∗*^*p* < 0.01 versus control group, ^*∗*^*p* < 0.05 versus control group).

### 3.7. Icariin Had a Strong Stimulative Effect on the PI3K/AKT Pathway of Human Nucleus Pulposus (Figure 7(a))

**Figure 7:**
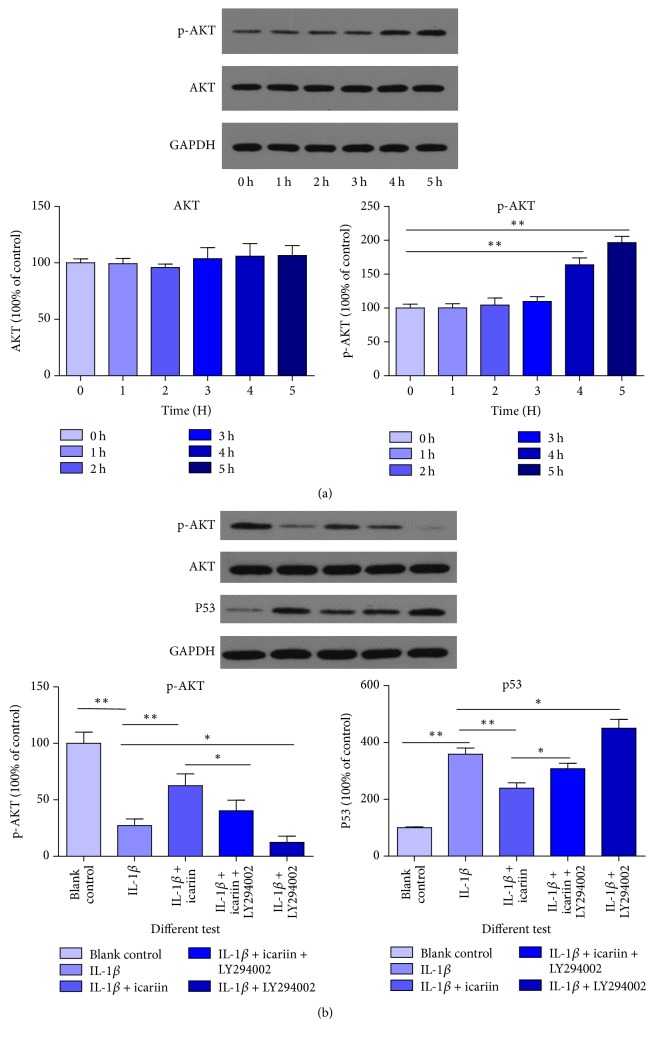
(a) Icariin had significant stimulative effects on the PI3K/AKT pathway when the reaction time reached 4 h and greater; results were detected by western blot of p-AKT (^*∗∗*^*p* < 0.01 versus control group). (b) IL-1*β* inhibited the PI3K/AKT pathway, but icariin attenuated this effect (^*∗∗*^*p* < 0.01 versus control group, ^*∗*^*p* < 0.05 versus control group). We think the PI3K/AKT pathway is partly involved in the protective effect of icariin through an antiapoptosis process.

Over time, the stimulative effects of icariin on the PI3K/AKT pathway became more evident. During the observation period, after the 4 h intervention, a remarkable stimulative effect of icariin on the PI3K/AKT pathway was evident. These results provided strong evidences that icariin had the ability to stimulate the PI3K/AKT pathway alone. What is more, the PI3K/AKT pathway was involved in the protective process (Figure 7(b)) (^*∗∗*^*p* < 0.01 versus control group, ^*∗*^*p* < 0.05 versus control group). AKT is a vital molecule in the PI3K/AKT pathway and p-AKT is biologically active in the same pathway. P53 is a protein downstream and is inhibited by activated AKT (p-AKT). This variation indicated an important effect of the PI3K/AKT pathway in the process.

## 4. Discussion

Inflammation is an important risk factor of intervertebral disc degeneration (IDD). Some inflammatory cytokines, such as IL-1*β*, IL-6, IL-10, and TNF-*α*, have been explored to be related to IDD [20–27]. Excessive deposition of inflammatory cytokines in the microenvironment of intervertebral disc contributes to degeneration of the intervertebral disc [28]. As a traditional Chinese medicine, icariin has a strong antioxidative [29–32] and anti-inflammatory effect [33–36] in many vital tissues; however, there were no studies reporting its effect in the intervertebral disc.

We have simulated an inflammatory microenvironment in human NP cells using IL-1*β*, an important inflammatory factor. We found cytotoxicity of IL-1*β* at a concentration of 20 ng/mL, which could induce apoptosis for human NP cells; thus we used this concentration for our experiment.

Icariin had a strong protective effect on IL-1*β* pretreated human NP cells. This anti-inflammatory effect was detected by the stabilization of mitochondrial membrane potential, apoptosis rate, apoptosis relative proteins, intracellular ROS rate, and imaging cells in an electron microscope. All of these results supported each other and indicated that icariin could protect against IL-1*β*-induced cell apoptosis and cellular instability. In many other areas, the similar function of icariin was observed, such as cerebral ischemia-reperfusion injury [37, 38], osteolysis and inflammatory response in bone [39], and lipopolysaccharide-induced brain dysfunction [40]. We conclude that icariin has an anti-inflammatory effect and, to our knowledge, this paper is the first one to point out that it may have potential application in NP cells and intervertebral disc degeneration.

In addition to the protect effect of icariin in an inflammatory environment, we explored possible mechanism behind this phenomenon. The PI3K/AKT pathway is important to and responsible for cell life cycle, proliferation, aging, survival, and apoptosis [41]. There are many literatures proposing that PI3K/AKT may be a target molecular signaling pathway in oncotherapy [42–44] which raises the importance of PI3K/AKT pathway. However, excessive activation of PI3K/AKT pathway may lead to neoplastic lesion [45]; appropriate activation is essential when this pathway is used in antiapoptosis. Our research exhibited that when cells were exposed to adverse environmental factors, the activation of PI3K/AKT pathway had positive meanings. Icariin's ability to stimulate the PI3K/AKT pathway has been pointed out many times and is related to antiapoptosis [9, 46], antioxidative stress [15], the promotion of differentiation [14] and sex [47], and the protective effect of ischemia reperfusion [48]. In our research, we observed significant stimulative effects on the PI3K/AKT pathway by icariin when the interaction time reached 4-5 h; therefore there are evidences to believe that the antiapoptosis effect of icariin is linked to this pathway. In subsequent experiments, we designed five groups to explore the protective effect of icariin and the role of the PI3K/AKT pathway in this process. All of the results supported that icariin had a strong protective effect on IL-1*β* pretreated human NP cells and that the PI3K/AKT pathway was, at least partly, involved in this protective effect. The apoptosis rate, ROS rate, JC-1 detection, and apoptosis-related proteins had similar results.

In summary, to our knowledge, this study is the first one to propose the anti-inflammatory effect of icariin on human NP cells. The present research provides a theoretical basis for icariin's application in the treatment of IDD.

## Figures and Tables

**Figure 4:**
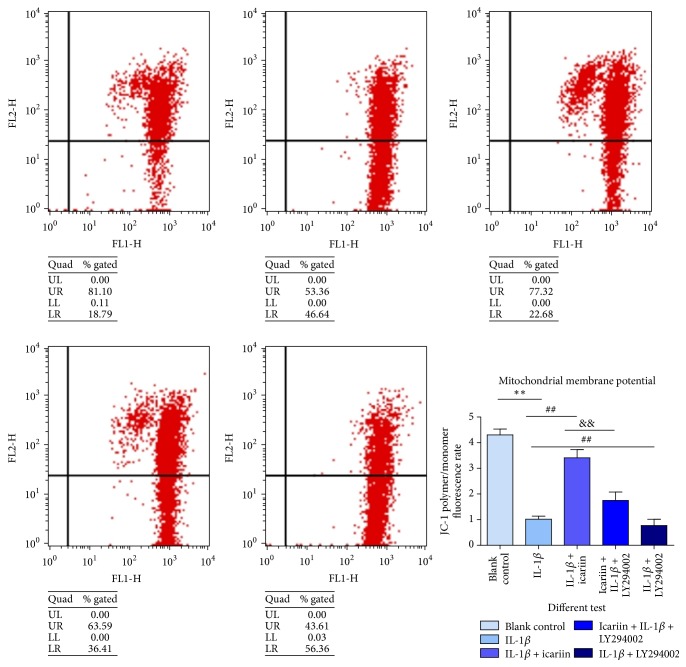
Given 20 *μ*g/mL of IL-1*β* for 48 h, human NP cells exhibited a decline in MMP. The group pretreated with icariin exhibited a stable mitochondrial membrane potential. When the PI3K/AKT pathway was blocked, this protective effect was weakened. There were significant statistical differences between groups, as labeled in the figure (^*∗∗*^*p* < 0.01 versus control group, ^##^*p* < 0.05 versus IL-1*β* group, and ^&&^*p* < 0.05 versus IL-1*β* + icariin group).

**Figure 6:**
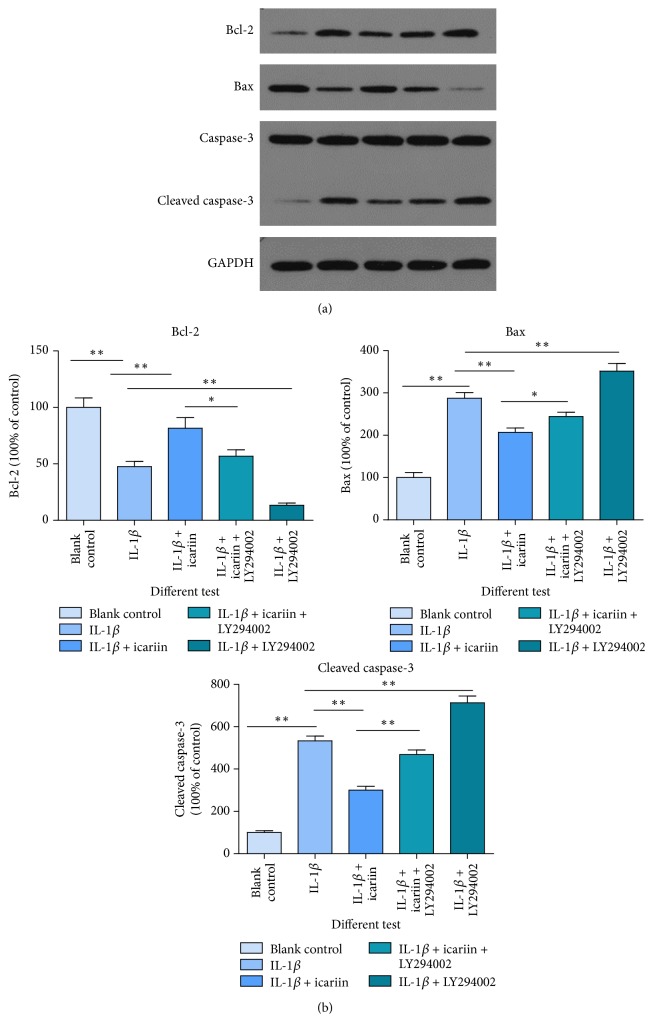
As shown above, IL-1*β* could induce apoptosis-related protein changes. (a) Bax and cleaved caspase-3 are two important apoptosis-promoter proteins and Bcl-2 is an antiapoptosis protein. An increase of Bcl-2 and a decline of bax and cleaved caspase-3, when pretreated with icariin, showed a protective effect of icariin. (b) The bar graph comes from (a) (^*∗∗*^*p* < 0.01 versus control group, ^*∗*^*p* < 0.05 versus control group). When the PI3K/AKT pathway was blocked by LY294002, this protective effect was diminished.
